# The effect of pH, electrolytes and temperature on the rhizosphere geochemistry of phytosiderophores

**DOI:** 10.1007/s11104-017-3226-9

**Published:** 2017-04-13

**Authors:** M. Walter, S. M . Kraemer, W. D. C. Schenkeveld

**Affiliations:** Department of Environmental Geosciences and Environmental Science Research Platform, University of Vienna, Althanstraße 14 (UZA II), 1090 Vienna, Austria

**Keywords:** Fe acquisition, Phytosiderophore, Deoxymugineic acid, Temperature, Electrolyte, pH, Rhizosphere, Metal mobilization, Adsorption, Liming

## Abstract

**Background and aims:**

Graminaceous plants are grown worldwide as staple crops under a variety of climatic and soil conditions. They release phytosiderophores for Fe acquisition (Strategy II). Aim of the present study was to uncover how the rhizosphere pH, background electrolyte and temperature affect the mobilization of Fe and other metals from soil by phytosiderophores.

**Methods:**

For this purpose a series of kinetic batch interaction experiments with the phytosiderophore 2′-deoxymugineic acid (DMA), a calcareous clay soil and a mildly acidic sandy soil were performed. The temperature, electrolyte concentration and applied electrolyte cation were varied. The effect of pH was examined by applying two levels of lime and Cu to the acidic soil.

**Results:**

Fe mobilization by DMA increased by lime application, and was negatively affected by Cu amendment. Mobilization of Fe and other metals decreased with increasing ionic strength, and was lower for divalent than for monovalent electrolyte cations at equal ionic strength, due to higher adsorption of metal-DMA complexes to the soil. Metal mobilization rates increased with increasing temperature leading to a faster onset of competition; Fe was mobilized faster, but also became depleted faster at higher temperature. Temperature also affected biodegradation rates of metal-DMA complexes.

**Conclusion:**

Rhizosphere pH, electrolyte type and concentration and temperature can have a pronounced effect on Strategy II Fe acquisition by affecting the time and concentration ‘window of Fe uptake’ in which plants can benefit from phytosiderophore-mediated Fe uptake.

**Electronic supplementary material:**

The online version of this article (doi:10.1007/s11104-017-3226-9) contains supplementary material, which is available to authorized users.

## Introduction

Iron (Fe) acquisition by plants grown on alkaline soils is limited by the low solubility and slow dissolution kinetics of iron-bearing minerals (Takagi 1976; Kraemer et al. 2006). Plants have developed Fe acquisition strategies to avoid Fe deficiency under such conditions. Strategy I plants (non-graminaceous species) upregulate ferric chelate reductase (FCR) activity and enhance exudation of protons and phenolic compounds into the rhizosphere to increase Fe availability. Strategy II plants (graminaceous plants) exude phytosiderophores (PS), which are chelating ligands with a high affinity for Fe. PS comprise a relatively small group of ligands including mugineic acid (MA) and its derivatives 3′-epi-hydroxymugineic acid (epi-HMA) and 2′-deoxymugineic acid (DMA) (Murakami et al. 1989). They form hexadentate complexes with Fe through binding with aminocarboxylate and hydroxycarboxylate functional groups (Kraemer 2004). In the rhizosphere PS can form soluble FePS complexes that can readily be taken up by graminaceous plants (Roemheld and Marschner 1986; Roemheld 1987). The majority of graminaceous plants release PS in diurnal pulses, with the highest exudation rates 2–6 h after the onset of light (Roemheld and Marschner 1986; Oburger et al. 2014). The diurnal pulse release is affected by light regime (Reichman and Parker 2007) and by temperature (Ueno and Ma 2009). Exudation of PS is strongly enhanced under conditions of Fe deficiency.

Upon exudation, PS participate in rhizosphere processes. These processes draw up a time and concentration ‘window of Fe uptake’ in which plants can benefit from PS-facilitated Fe acquisition (Schenkeveld et al. 2014b). This window is constrained by processes and factors that either increase or decrease Fe mobilization. Known processes and factors that increase Fe mobilization include a high rate and increased duration of PS exudation, a high solubility of soil Fe minerals and fast Fe release rates from the soil. Processes that decrease Fe mobilization include adsorption of PS ligands and metal-PS complexes, degradation of the PS ligand and competitive complexation of metals other than Fe. The size of the Fe uptake window strongly varies among soils (Schenkeveld et al. 2014a), suggesting that it depends on soil properties.

PS can mobilize Fe from Fe(hydr)oxide minerals (Reichard et al. 2005) and Fe bound to humic substances (Cesco et al. 2002). Fe dissolution of Fe(hydr)oxide minerals by PS is inversely correlated to the crystallinity of the Fe(hydr)oxide phase (Hiradate and Inoue 1998). Furthermore, adsorption of inorganic anions like carbonate and phosphate onto such minerals decreases the rate of Fe mobilization by PS (Watanabe and Matsumoto 1994);

Apart from Fe(III), PS can also chelate and mobilize other metals from soil, including Cd, Co, Cu, Mn, Ni and Zn (Awad et al. 1999; Zhang 1993; Schenkeveld et al. 2014a, b; Takagi 1976; Treeby et al. 1989). These metals compete with Fe for complexation by PS. This competition increases the rate by which free PS becomes depleted from soil solution and causes displacement of Fe from FePS complexes. Hence competition leads to a reduction in size of the window of Fe uptake (Schenkeveld et al. 2014b). For an uncontaminated calcareous soil from Santomera, Spain, Cu was shown to be the principal competing metal for complexation (Schenkeveld et al. 2014b; Schindlegger et al. 2015).

PS are subject to biodegradation by microorganisms (Shi et al. 1988);(Oburger et al. 2016)), which use it as a carbon source (Takagi et al. 1988). Microorganisms decreased the detectable amount of PS in growing media and induced Fe deficiency in Strategy II plants (von Wiren et al. 1993). In soils, biodegradation caused a faster depletion from solution of the free PS ligand than of certain metal-PS complexes (e.g. CuDMA and NiDMA) (Schenkeveld et al. 2014b).

Also, adsorption of metal-PS complexes to the soil solid phase lowers their concentration in soil solution. For FeDMA, CuDMA, NiDMA and ZnDMA (0 - 100 μM) and the free DMA ligand (0 - 1000 μM) adsorption to soil proved to be linear and partly reversible (Walter et al. 2016). Adsorption kinetics were shown to be fast in comparison to metal mobilization kinetics. Among the DMA species examined, NiDMA had the strongest tendency to adsorb and CuDMA the weakest, and adsorption of metal-PS complexes correlated most strongly with the ratio of a clay content over soil organic matter content (Walter et al. 2016).

Graminaceous plants like wheat, barley, maize and rice are grown worldwide as staple crops under diverse climatic and soil conditions. These conditions are likely to affect the reactivity of PS in the rhizosphere and hence the window of Fe uptake. In the present study we examine the influence of pH, electrolyte type and concentration, and temperature on the size of this window.

pH influences the soil solution speciation of PS in multiple ways. It determines the protonation state and coordination of metal-PS complexes (von Wiren et al. 2000) and affects the free activity of metals in soil solution (Schenkeveld et al. 2015). Hence, the soil pH will co-determine to what extent PS ligands can complex and mobilize iron from a soil in order to facilitate Fe uptake. Equilibrium modelling suggests that in soils with a low pH, FePS complexes dominate PS speciation, whereas at alkaline soil pH, NiPS and CuPS are dominant (Reichman and Parker 2007; Schenkeveld et al. 2014a). In soil interaction experiments, Schenkeveld et al. (2014a) observed that PS mainly mobilized Fe from an acidic soil (pH = 4.5), whereas from most soils with circum-neutral pH, mainly competing elements were mobilized. However, the examined soils also differed in other properties than pH. The influence of soil-pH on metal mobilization by PS has not yet been examined in a targeted way, in which the contents of soil reactive compounds relevant for adsorption, and the contents of metals relevant for complexation were kept constant. The rhizosphere and soil pH are not exclusively determined by the native soil-pH, but also by natural processes like rhizosphere acidification by plants (Guo et al. 2010) and anthropogenic processes like liming (Tyler and Olsson 2001) and fertilization (Barak et al. 1997). Soil liming can change metal availability (Tyler and Olsson 2001) and over-application of lime may cause adverse effects on plant biomass production (Fageria et al. 1995).

Plants can be exposed to high salt concentrations in naturally occurring saline soils (Volkmar et al. 1998), and in soils that have received high anthropogenic salt input e.g. through irrigation (Pitman and Lauchli 2002) or de-icing salt application (Davison 1971). In a hydroponic study, it was shown that PS exudation by Zn-deficient wheat increased with the electrolyte concentration of the nutrient solution (Daneshbakhsh et al. 2013). Oburger et al. (2014) observed in a pot trial with wheat that the PS exudation rate may increase with soil salinity. Furthermore, increased NaCl concentration was shown to depress the mobilized concentration of labeled Fe from a calcareous soil by PS exuded from barley (Awad et al. 1988), but the authors could not explain this observation. The effect of electrolyte type on metal mobilization by PS from soil has not yet been examined.

Soil temperature varies in daily and annual cycles and depends on the climatic and geographic location of the soil profile (Zheng et al. 1993). No studies on the influence of temperature on PS reactivity have been reported yet. However, for the microbial siderophore desferioxamine B (DFOB) goethite dissolution rates were shown to increase with temperature, while adsorption of the DFOB ligand proved to be largely temperature independent. (Cocozza et al. 2002).

Based on the concepts discussed above we tested the following hypotheses:

- In accordance with the aforementioned modelling studies, we hypothesize that increasing the soil pH by lime application will enhance the mobilization of competing metals by PS and will therefore reduce the size of the window of Fe uptake.
- We hypothesize that the electrolyte affects the electrostatic interaction between reactive soil surfaces and PS-species, and thereby adsorption of PS-species and metal mobilization by PS. This in turn affects the size of the window of Fe uptake.
- We hypothesize that the rates of metal mobilization from soil by PS increase with increasing temperature, and, as a consequence, that Fe displacement from FePS complexes will set in earlier, reducing the size of the window of Fe uptake.

To address the identified knowledge gaps regarding the influence of rhizosphere pH, electrolyte and temperature on Strategy II Fe acquisition, and to test the aforementioned hypotheses we have conducted a series of interaction experiments with soils and the PS 2′-deoxymugineic acid (DMA) in which the soil pH (by liming) and its interplay with copper, the type and concentration of the electrolyte, and the reaction temperature were varied. Experimental results have been interpreted in the framework of the conceptual ‘window of Fe uptake’ model.

## Materials and methods

### Materials

*Soil* – A calcareous clay soil was collected from a site in Santomera (Murcia, Spain) and a mildly acidic sandy soil was collected from a site in Siebenlinden (Lower Austria, Austria). For both soils the top layer (0–20 cm) was sampled. These soils have been used previously in studies on Fe deficiency and both Strategy I and Strategy II plants grew chlorotic on Santomera soil (Oburger et al. 2014; Schenkeveld et al. 2010). Before use, the soils were air-dried and sieved (2 mm mesh size).

Batches of the Siebenlinden soil were either treated with lime (up to 0.5 mass percent) or not. In order to keep the contents of reactive soil constituents (SOM, clay mineral, (oxy)hydroxide minerals) the same for all batches, quartz sand was added up to 5 (not limed) and 4.5 (limed) mass percent. The DTPA-extractable Cu content of Siebenlinden soil (Table 1) is much lower than added amount of DMA in these experiments so that it does not play a substantial role in competing for complexation by the DMA ligand. In order to examine the influence of pH and liming on competitive complexation of Cu and Fe mobilization by DMA, the Cu content of the soil was raised by Cu application. To batches of both the limed and the unlimed Siebenlinden soil, either 0 or 5 mg/kg Cu was applied as Cu citrate. Four batches of Siebenlinden soil were created: 1) 0% CaCO_3_ and 0 mg kg^−1^ Cu added, 2) 0.5% CaCO_3_ and 0 mg kg^−1^ Cu added, 3) 0% CaCO_3_ and 5 mg kg^−1^ Cu added, and 4) 0.5% CaCO_3_ and 5 mg kg^−1^ Cu added. To facilitate mixing and equilibration, the Siebenlinden soil batches were subjected to 5 wetting-drying cycles at 30–40 °C in the course of 2 months. Water was repeatedly added and mixed with the soil up to a soil solution ratio (SSR) of 5. Citrate has a half-life in the order of a few hours in soil (Oburger and Jones 2009); therefore the citrate that had been added with the Cu was assumed to be completely degraded after 2 months. Selected soil properties of Santomera soil and the Siebenlinden batches are presented in Table 1.

**Table 1:** Selected soil properties of Santomera and Siebenlinden soil including carbonate and Cu amended soil batches (note: quartz sand was added as needed to keep the mass of reactive soil constituents constant in all amended soils)

	Santomera	Siebenlinden Original soil	Amended Siebenlinden I: 5% Quartz 0% CaCO_3_ 0 mg kg^−1^ Cu	Amended Siebenlinden II: 4.5% Quartz 0.5% CaCO_3_ 0 mg kg^−1^ Cu	Amended Siebenlinden III: 5% Quartz 0% CaCO_3_ 5 mg kg^−1^ Cu	Amended Siebenlinden IV: 4.5% Quartz 0.5% CaCO_3_ 5 mg kg^−1^ Cu
Region	Murcia	Lower Austria				
Country	Spain	Austria				
Soil classification	Entisol	Cambisol				
pH CaCl_2_	7.5	4.5	4.5	6.8	4.5	6.8
Clay (g kg^−1^)	300	100				
SOM (g kg^−1^)	15	26				
CaCO_3_ (g kg^−1^)	500	0	0	5.3	0	5.3
Amox Fe (g kg^−1^)	0.5	2.8				
DCB Fe (g kg^−1^)	10.2	10.6				
DTPA- Extraction:						
Fe (mg kg^−1^)	4.9	47	56	19	52	17
Cu (mg kg^−1^)	1.6	0.1	0.2	0.1	1.9	1.3
Ni (mg kg^−1^)	0.3	0.1	0.1	0.0	0.1	0.0
Zn (mg kg^−1^)	0.5	1.3	0.6	0.2	0.6	0.2
Co (mg kg^−1^)	0.0	0.0	0.0	0.0	0.0	0.0
Mn (mg kg^−1^)	3.1	6.5	5.3	2.4	9.9	2.3

*DMA* – ammonium DMA salt was synthesized in accordance with Namba et al. (2007). The DMA salt was over 95% pure in accordance with NMR analysis. The salt readily dissolved in water.

Analytical grade chemicals and ultra-pure water were used for preparing solutions.

## Experiments

### General procedure

Kinetic experiments - Soil and DMA solution interacted in 50 ml polypropylene centrifuge tubes (Greiner bio one) in a SSR of 1 (*w*/*v*). 10 mM CaCl_2_ was used as default background electrolyte. To examine the influence of microbial activity, treatments with and without addition of sodium azide or Bronopol (2-bromo-2-nitropropane-1,3-diol) as sterilant were included. It was demonstrated that during the initial lag phase these sterilants did not influence the observations relative to experiments without sterilants, but they suppressed biological activity throughout the experiment (Schenkeveld et al. 2014b and SI-Fig. 4). Blank treatments without addition of DMA were also included. Samples were pre-equilibrated with electrolyte solution and sterilant (when required) for 2 days at 90% of the final solution volume prior to administration of the DMA treatment. After application of the treatment, samples were again placed in an end-over-end shaker rotating at 18 rpm in the dark at 20 (±1) °C (except in the temperature experiment). Sampling was done after 0.25, 0.5, 1, 2, 4, 8, 24, 48, 96, 168 h. Samples were centrifuged for 3 min at 4500 rpm and filtered over 0.45 μm cellulose acetate filters (Whatman Aqua 30/0.45 CA).

Adsorption equilibrium experiments – Adsorption experiments were done with Santomera soil and FeDMA and CuDMA at a SSR of 1. The soil was pre-equilibrated for two days prior to application of the treatment at 90% of the final solution volume. FeDMA and CuDMA were added to a concentration of 1, 5, 25, 50 and 100 μM, and sampled after 2 h of interaction. This interaction time was shown to be sufficient for establishing adsorption equilibrium (Walter et al. 2016). No sterilant was added, because metal-DMA complex concentrations had been shown to be unaffected by biodegradation within this interaction time (Walter et al. 2016). Samples were either shaken in an end-over-end shaker (ionic strength experiment) or in a horizontal shaker (temperature experiment). Samples were centrifuged at 4500 rpm and filtered over 0.45 μm cellulose acetate filters (Whatman Aqua 30/0.45 CA). The pH of the filtrates was measured and the filtrates were further analysed.

### Soil pH experiment

The influence of soil pH and liming on metal mobilization by DMA was examined as a function of time. Cu content was also included as a variable in this experiment to examine the pH dependent competitive effect of Cu on Fe mobilization by DMA. Four batches of Siebenlinden soil that had been limed to a different extent (0 and 0.5 mass percent) and had received different amounts of Cu (0 or 5 mg kg^−1^ Cu) interacted with a 30 μM DMA solution. 0.2 g l^−1^ Bronopol was applied as a sterilant.

### Electrolyte experiment

The influence of electrolyte concentration and composition on metal mobilization by PS was examined as a function of time by interacting Santomera soil with solutions containing 100 μM DMA and various background electrolytes: ultra-pure water ((UPW); no added electrolyte), 2, 10 and 100 mM CaCl_2_ and 300 mM NaCl. The latter two treatments impose the same ionic strength (*I* = 0.3 M), but differ in the valence of the cation in order to examine the effect of electrolyte composition in addition to the effect of ionic strength. Bronopol was used as sterilant, because it is non-ionic and therefore its effect on ionic strength can be neglected, which is particularly important for the low ionic strength treatments (UPW and 2 mM CaCl_2_). The pH varied by up to 0.9 pH units in CaCl_2_ and 0.5 pH units in NaCl treatments relative to UPW treatment (SI-Table 1). Additionally, adsorption isotherms were determined for adsorption of FeDMA and CuDMA to Santomera soil for all aforementioned electrolytes. Samples interacted in an end-over-end shaker rotating at 18 rpm.

### Temperature experiment

The influence of temperature on metal mobilization from soil by PS was examined as a function of time by interacting Santomera soil with a 100 μM DMA solution at 7.5 (± 1.5), 20 (±1), 35 (±1) and 60 (±1) °C. 2 g l^−1^ NaN_3_ was applied in treatments requiring a sterilant. Samples were horizontally shaken in an incubator shaker (Excella E24, New Brunswick Scientific; 7.5, 35 and 60 °C) and a table shaker (IKA-Werke KS501; 20 °C) at 100 rpm. Additionally, adsorption isotherms were determined for FeDMA and CuDMA at 7.5 (± 1.5), 20 (±1), 35 (±1) °C.

### Analysis

Metal concentrations (Fe, Cu, Ni, Zn, Co, Mn) were measured by ICP-OES (Perking Elmer, Optima 5300-DV). Samples were acidified with HNO_3_ before analysis. Metal-DMA complex concentrations were calculated as the difference in metal concentration between the DMA treatment and the corresponding blank. This is also referred to as ‘mobilized metal concentrations’ in this publication.

## Results

### Effect of soil pH

Lime application (0.5 mass percent) to Siebenlinden soil increased the pH from 4.5 to 6.8. Addition of 5 mg kg^−1^ Cu did not affect the soil pH. Liming up to 0.5 mass percent and the resulting increase in pH consistently decreased metal availability as expressed in the diethylenetriaminepentaacetic acid (DTPA)-extractable contents of Fe, Cu, Ni, Zn and Mn (Table 1). Additional lime application up to 5 mass percent was tested, but only resulted in a minor further increase in pH, to 7.1; metal availabilities were also comparable in 0.5 and 5 mass percent limed treatments (data not shown). Both in the treatments with and without Cu addition, Fe availability decreased by approximately a factor three as a result of liming. Liming had a smaller effect on Cu availability. The 5 mg kg^−1^ Cu addition strongly increased the Cu availability of the soil. The DTPA-extractable Cu content increased by approximately one fourth to one third of the amount of Cu added, suggesting that not all added Cu remained bioavailable. In the non-limed treatments, Cu addition also led to an increase in DTPA-extractable Mn content from 5.3 to 9.9 mg kg^−1^; the reason for this is unclear.

**Fig. 1:**
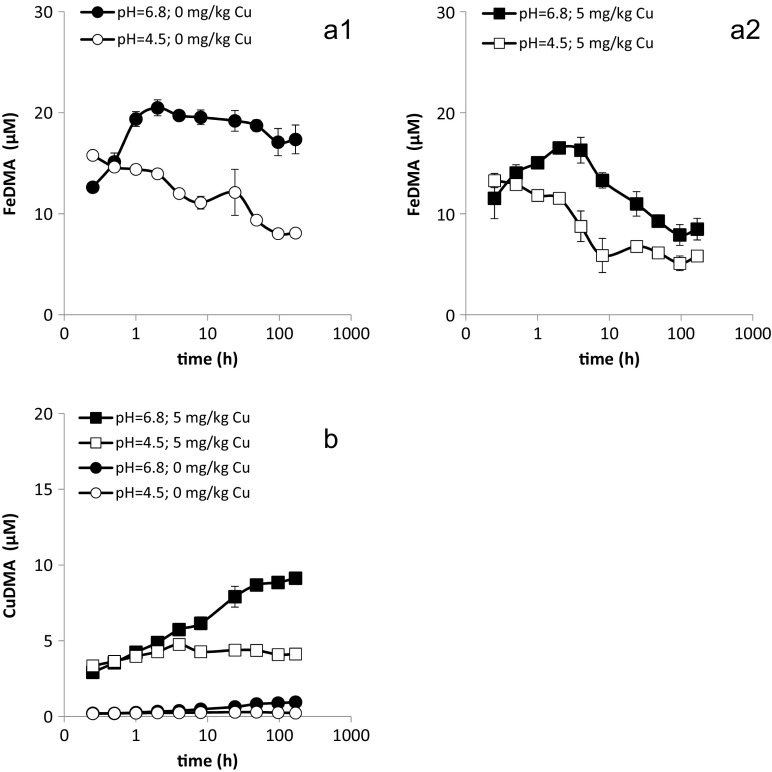
Effect of liming and Cu content on the mobilization of a1) Fe (without Cu amendment) a2) Fe (with 5 mg kg^-1^ Cu amendment) and b) Cu (with and without 5 mg kg^-1^ Cu amendment) from Siebenlinden soil by 30 μmol l^−1^ DMA as a function of time (SSR = 1; 10 mM CaCl_2_). The acidic Siebenlinden soil (pH = 4.5) does not contain CaCO_3_ and was amended with 0.5 mass percent of CaCO_3_ to a pH of 6.8. Bronopol has been used as a sterilant. Error bars indicate standard deviations

**Fig. 2:**
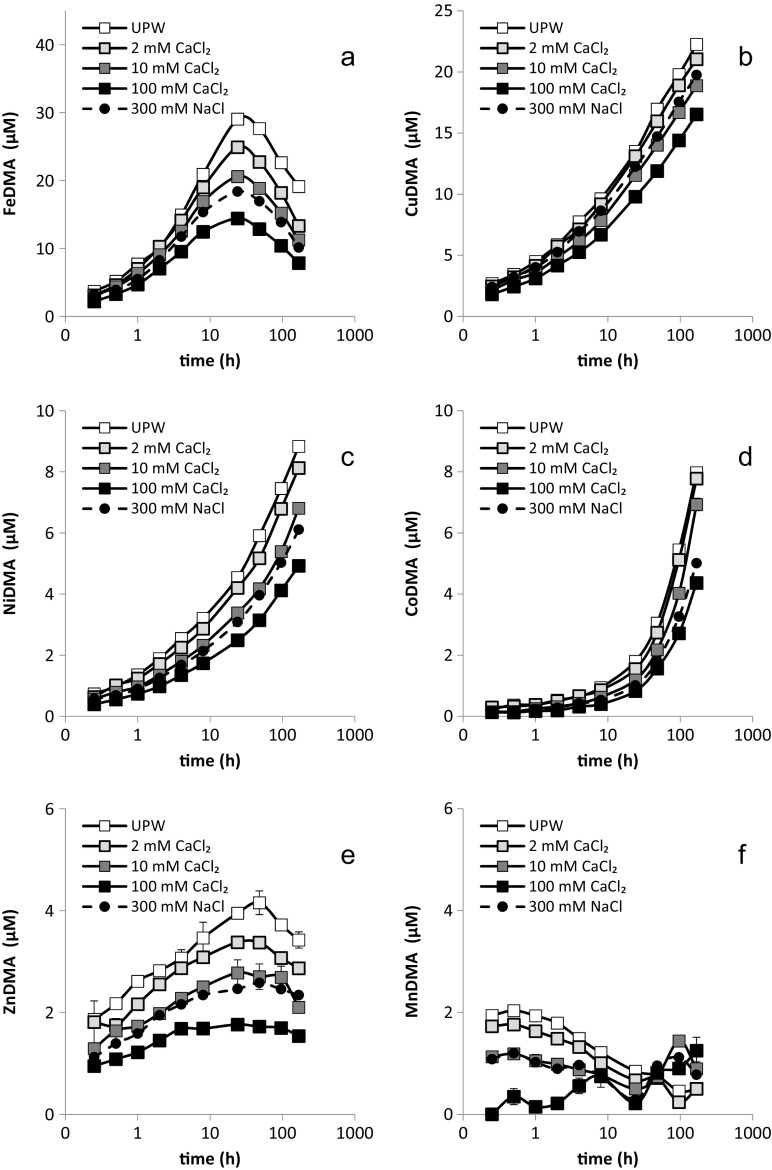
Mobilization of a) Fe, b) Cu, c) Ni, d) Co, e) Zn and f) Mn from by Santomera soil by a 100 μM DMA solution (SSR = 1; 0.2 g l-^1^ Bronopol) for various background electrolytes. Error bars indicate standard deviations. The 100 mM CaCl_2_ and 300 mM NaCl treatments have the same ionic strength (IS = 300 mM)

**Fig. 3:**
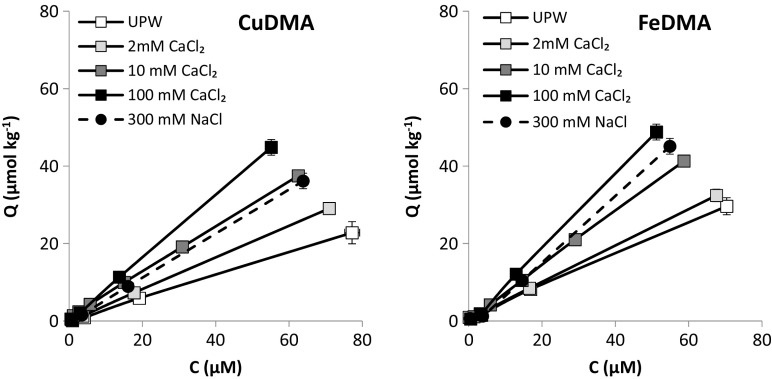
Adsorption isotherms for a) CuDMA and b) FeDMA to Santomera soil for various electrolytes. Error bars indicate standard deviations. The 100 mM CaCl_2_ and 300 mM NaCl treatments have the same ionic strength (300 mM)

Only Fe and Cu were mobilized in concentrations over 1 μM from the Siebenlinden soil batches by 30 μM DMA application (Fig. 1); for reasons of clarity, mobilized Fe concentrations are presented in separated panels for treatments without Cu addition (Fig. 1a1) and treatments with Cu addition (Fig. 1a2). Zn was temporarily mobilized in sub-micromolar concentrations in the limed soils for up to 2 h and mobilization was not influenced by addition of 5 mg kg^−1^ Cu to soil (SI-Fig. 1). In the non-limed treatments (pH 4.5), considerable Mn concentrations, up to almost 190 μM, were found in solution, both in the blank and the DMA treatments (data not shown). No differences in Mn concentration between the blank and the DMA treatments were observed, and equilibrium modelling predicted no MnDMA complex formation in relevant concentrations under these conditions (SI-Fig. 2). Also Zhang (1993) had concluded that Mn is not efficiently chelated by DMA, neither in a calcareous soil (pH 8.1), nor in a slightly acidic soil (pH 5.5). Potential formation of CaPS as a result of lime amendment was examined by equilibrium modelling. For this purpose, DMA (30 μM) speciation was modelled as a function of pH under atmospheric CO_2_ partial pressure (400 ppm), in presence of 0.1 M Ca; the calcium carbonate mineral aragonite (which is more soluble than calcite) was allowed to precipitate (SI-Fig. 2b). Modelling results demonstrate that Ca is not a relevant metal with respect to competing with Fe for complexation by the PS ligand in any soil, with or without lime amendment. Even in absence of competing metals no relevant concentrations of CaDMA form at low pH (>6.5); at higher pH values, the equilibrium concentration of the free ligand still exceeds that of CaDMA by more than a factor 20. Elevated CO_2_ partial pressure in the rhizosphere will increase the carbonate (CO_3_^2−^(aq)) concentration and lower the Ca concentration, further decreasing the relevance of Ca as competing metal. Metal mobilization by DMA in corresponding treatments with 0.5 and 5 mass percent lime was almost identical (SI-Fig. 3), therefore only the results for 0.5 mass percent lime are shown in Fig. 1.

In treatments without Cu application, initial Fe mobilization was faster at pH 4.5 (Fig. 1a1) and the mobilized Fe concentration gradually declined from 15.8 μM after 0.25 h to 8.1 μM after 168 h. In the limed treatment (pH 6.8), the mobilized Fe concentration first increased from 12.6 μM after 0.25 h to 20.5 μM after 2 h, after which it remained more or less constant (17.8 μM after 168 h). Mobilized Fe concentrations in the non-limed treatment with Cu application developed similarly as in the corresponding treatment without Cu application, gradually declining from 13.3 μM (0.25 h) to 5.8 μM (168 h). They were, however, consistently lower in the treatment with Cu addition. In the limed treatment with Cu application, mobilized Fe concentrations first increased from 11.5 μM (0.25 h) to 16.5 μM (2 h) and then gradually decreased to 8.5 μM (168 h). Up to 0.5 h, there was no difference in mobilized Fe concentrations between the limed treatments with and without Cu application.

Mobilized Cu concentrations were considerably larger from the soil batches that had received Cu application (Fig. 1b). Initially, Cu mobilization was comparable for corresponding limed and non-limed treatments, but eventually mobilized Cu concentrations in the limed treatment became larger. For the treatments without Cu application, mobilized Cu concentrations in the non-limed soil remained approximately constant (0.2 μM), whereas in the limed treatment it consistently increased to 0.9 μM. In the non-limed treatments with Cu application, the CuDMA concentration first increased from 3.3 μM (0.25 h) to 4.8 μM (4 h), after which it remained approximately constant. In the limed treatment with Cu application, the CuDMA concentration increased throughout the experiment from 2.9 μM (0.25 h) to 9.1 μM (168 h).

**Fig. 4:**
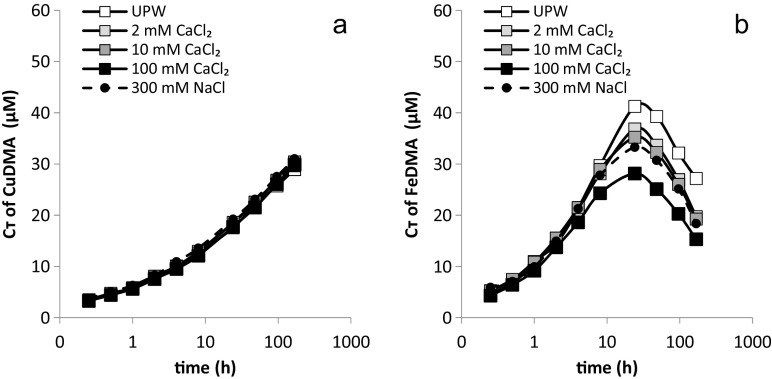
The total concentration (C_T_, including soluble and adsorbed concentrations) of a) FeDMA and b) CuDMA in Santomera soil calculated for all examined electrolyte types and concentrations. SSR = 1; 0.2 g l^−1^ Bronopol was added as sterilant. Error bars indicate standard deviations

The effect of liming on microbial degradation of metal-DMA complexes was examined with soil batches without Cu application. The lag phase until differences in FeDMA and CuDMA concentrations between treatments with and without sterilant addition occurred was similar: between 8 and 24 h (SI-Fig. 4). In the limed treatment without sterilant, FeDMA and CuDMA concentrations decreased below the LOQ after 48 h compared to 96 h in the non-limed treatment, indicating that biodegradation rates of the metal-DMA complexes were increased by lime application.

The DTPA-extractable metal contents of the Siebenlinden soil batches reflect mobilization by DMA only to a limited extent metal. The larger available Fe content in the non-limed batches was reflected in the rate by which the maximum mobilized Fe concentration was reached, but not in the maximum mobilized Fe concentration. The latter was in fact higher in the limed batches than in the corresponding non-limed batches from 0.5 h onward. The higher DTPA-extractable Cu content in the soil batches that had received Cu application was reflected in higher mobilized Cu concentrations by DMA. However, DMA eventually mobilized more Cu from the limed soil batches compared to the corresponding non-limed batches, although Cu availability was lower in the limed batches. For the non-limed treatments, the largest additionally mobilized Cu concentration related to the Cu amendment amounted 3.9 μM, corresponding to 5% of the Cu amendment, and 13% of the DTPA-extractable Cu in the Cu amended treatment. For the limed treatments, the largest additionally mobilized Cu concentration amounted 8.2 μM, corresponding to 10% of the Cu amendment, and 43% of the DTPA-extractable Cu in the Cu amended treatment.

### Effect of type and concentration of the electrolyte

Metal mobilization by DMA from Santomera soil was strongly affected by the type and concentration of the electrolyte. The differences in pH as a result of the application of different electrolyte types and concentrations (SI-Table 1) were too small to induce the observed effects. Fe and Cu were mobilized to the largest extent, followed by Ni and Co, while mobilized Zn and Mn concentrations were relatively small. Throughout the experiment and for all examined metals, mobilized concentrations were smaller at higher CaCl_2_ background concentrations (Fig. 2); hence differences in concentration were largest between the UPW and the 100 mM CaCl_2_ treatments. The Ca and Na concentrations measured in the sampled solutions deviated somewhat from the applied concentrations (SI-Table 1) as a result of mobilization of soil-native cations and sorption to the soil solid phase. For electrolyte solutions of equal ionic strength (0.3 M), mobilized metal concentrations were consistently higher for the NaCl electrolyte compared to the CaCl_2_ electrolyte. For each metal individually, the time point at which the maximum solution concentration was reached was independent of the type and concentration of the electrolyte.

The mobilized Fe concentration first increased, reached a maximum after 24 h and then decreased (Fig. 2a) as a result of Fe displacement from the FeDMA complex by competing metals. This development corresponds with results previously reported by Schenkeveld et al. (2014b). Differences in mobilized Fe concentrations among the treatments showed a similar development; initially they were small, they increased until FeDMA concentrations reached a maximum and then decreased again. The largest difference in mobilized Fe concentrations was observed between the 100 mM CaCl_2_ and the UPW treatments after 24 h, corresponding to a factor two (14.5 μM and 29.0 μM, respectively). Mobilized Fe concentrations in the 300 mM NaCl treatment were consistently in between the mobilized concentrations in the 10 and 100 mM CaCl_2_ treatments.

The mobilized concentrations of important competing metals like Cu, Ni and Co (Fig. 2b-d) increased throughout the experiment in all treatments. The differences in mobilized concentrations between the electrolyte treatments also increased; after 168 h the difference between the 100 mM CaCl_2_ and the UPW treatments amounted a factor 1.4 for Cu (16.5 μM and 23.3 μM, respectively), a factor 1.8 for Ni (4.9 μM and 8.8 μM, respectively), and a factor 1.8 for Co (4.5 to 8.0 μM, respectively). Furthermore, the solution concentration ratios between different background electrolyte treatments remained largely constant throughout the experiment. Mobilized Cu concentrations in the 300 mM NaCl treatment were in between the concentrations in the 2 and 10 mM CaCl_2_ treatments, whereas mobilized Ni and Co concentrations in the 300 mM NaCl treatment remained in between their respective mobilized concentrations in the 10 and 100 mM CaCl_2_ treatments. The concentration ratios between the 300 mM NaCl and 100 mM CaCl_2_ treatments were however more constant among the metals (1.23–1.26) than the concentration ratios between the 10 mM CaCl_2_ and 100 mM CaCl_2_ treatments (1.19–1.49), suggesting that the effect of CaCl_2_ concentration varied more strongly among the metals than the effect of the cation in the electrolyte. An overview of all concentration ratios for CuDMA, NiDMA and CoDMA is presented in SI-Table 3.

Zn mobilization developed similarly as Fe mobilization; after an initial increase in Zn concentration, a maximum was reached after 24 to 48 h, followed by a decline in Zn concentration (Fig. 2e). The concentration difference between the 100 mM CaCl_2_ and UPW treatments after 48 h amounted 2.5 μM corresponding with a factor 2.5 (1.7 μM to 4.2 μM, respectively). Mobilized Mn concentrations reached a maximum after 1 h and the concentration difference between the 100 mM CaCl_2_ and UPW treatment amounted 1.8 μM corresponding with a factor 7 (0.3 μM to 2.1 μM, respectively) (Fig. 2f). Hence, on a relative scale, Mn mobilization was most strongly affected by ionic strength. Mobilized Zn and Mn concentrations in the 300 mM NaCl treatment were comparable with their respective concentrations in the 10 mM CaCl_2_ treatment.

Adsorption isotherms for FeDMA and CuDMA to Santomera soil proved linear over the concentration range examined (Fig. 3), as previously observed by Walter et al. (2016). The slope of the isotherms increased with increasing CaCl_2_ concentration, from 0.42 (UPW) to 0.95 (100 mM CaCl_2_) for FeDMA, and from 0.3 (UPW) to 0.81 (100 mM CaCl_2_) for CuDMA (Table 2). CuDMA adsorption was consistently smaller than FeDMA adsorption for all electrolytes. The type of electrolyte also affected adsorption. The slopes of the adsorption isotherms were larger for 100 mM CaCl_2_ than for 300 mM NaCl: 0.95 and 0.81, respectively, for FeDMA, and 0.81 and 0.57, respectively, for CuDMA. For CuDMA, the slope of the isotherm for the 300 mM NaCl treatment was also lower than for the 10 mM CaCl2 treatment (0.61).

**Table 2:** Linear adsorption isotherms for CuDMA and FeDMA to Santomera soil for various electrolytes (see also Fig. 3)

Species	electrolyte	Linear fit isotherm	R^2^
CuDMA	UPW	0.30*C	1.000
	2 mM CaCl2	0.41*C	1.000
	10 mM CaCl2	0.61*C	0.998
	100 mM CaCl2	0.81*C	1.000
	300 mM NaCl	0.57*C	1.000
FeDMA	UPW	0.42*C	0.998
	2 mM CaCl2	0.48*C	0.998
	10 mM CaCl2	0.71*C	1.000
	100 mM CaCl2	0.95*C	0.999
	300 mM NaCl	0.81*C	1.000

By means of these linear adsorption isotherms, the total FeDMA and CuDMA complex concentrations in suspension could be calculated for each time point of the kinetic experiments, under the assumption that adsorption equilibrium was reached instantaneously and preserved throughout the experiment. This was done using Eq. (1).

1 $$ {\mathrm{C}}_{\mathrm{T}}={\mathrm{C}}_{\mathrm{S}}+{\mathrm{C}}_{\mathrm{L}}={\mathrm{C}}_{\mathrm{L}}\bullet \left(1+\upalpha \bullet \mathrm{SSR}\right) $$

in which C_T_ is the total concentration of metal-DMA complex in suspension, C_S_ is the metal-DMA complex concentration adsorbed to the soil solid phase, C_L_ is the metal-DMA concentration in solution and α is the slope of the respective adsorption isotherm. The calculated total FeDMA and CuDMA concentrations for the various electrolyte treatments are presented as a function of time in Fig. 4. For CuDMA (Fig. 4b), the variation in total concentrations among the electrolyte treatments was very small throughout the experiment (the standard deviation remained below 1 μM). This implies that electrolyte type and concentration did not affect the rate at which Cu was complexed by DMA, but only the net-rate by which Cu was mobilized into solution, resulting from differences in partitioning between the solid and solution phase. Up until 4 h, the variation in total FeDMA (Fig. 4a) concentration among the electrolyte treatments was equally small, but from then on it started to increase, reaching differences up to 14.2 μM after 48 h between the UPW and the 100 mM CaCl_2_ treatment. The variation became particularly large from 24 h onward when competitive displacement of Fe from the FeDMA complex strongly affected the FeDMA concentration. Walter et al. (2016) recently showed that desorption of FeDMA is incomplete, and Schenkeveld et al. (2017) suggested that therefore the initial adsorption equilibrium partitioning does no longer apply once the FeDMA solution concentration starts to decline as are result of competitive Fe displacement. Hence, the total FeDMA concentration cannot be calculated with Eq. 1 once the FeDMA concentration starts to decline, because the adsorption isotherms do no longer apply.

### Effect of temperature

**Fig. 5:**
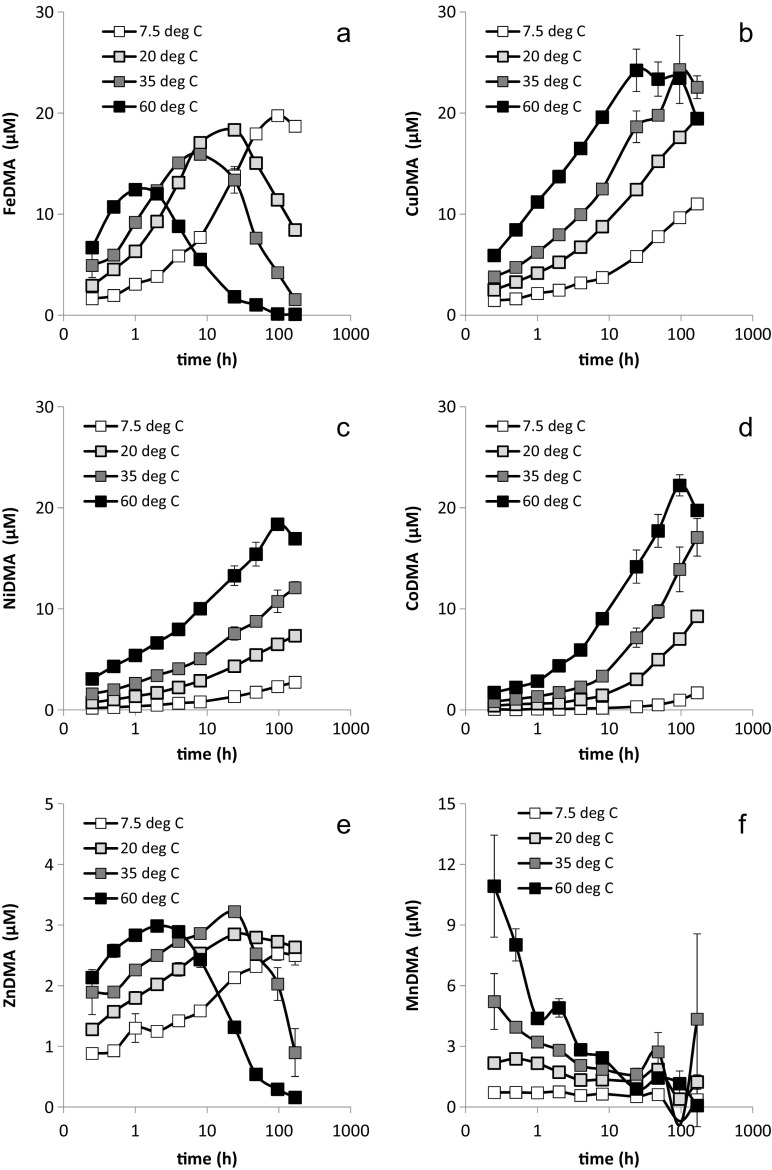
Mobilization of a) Fe, b) Cu, c) Ni, d) Co, e) Zn and f) Mn from Santomera soil by a 100 μM DMA solution (SSR = 1; 10 mM CaCl_2_; 2 g l^−1^ sodium azide) at various temperatures. Error bars indicate standard deviations

First the results for the treatments with sterilant addition are considered. For all examined metals, the (initial) mobilization rate from Santomera soil by DMA increased with increasing temperature (Fig. 5). This implies a faster depletion of the free DMA ligand. Hence for metal-DMA complexes susceptible to competitive displacement of the metal (particularly FeDMA and ZnDMA), the maximum concentration was reached after a shorter interaction time and the time-span the solution concentrations of the corresponding metals were elevated decreased with increasing temperature. The effect of temperature on the maximum soluble concentration depended on the metal-DMA complex.

Fe mobilization by DMA followed the same basic trend as in the electrolyte experiment: initially the FeDMA concentration increased, a maximum was reached, after which it decreased due to competitive displacement of the Fe. An increase in temperature caused a shift in mobilization maximum towards shorter interaction times. Furthermore, the maximum FeDMA concentration consistently decreased with increasing temperature from 19.8 μM after 96 h at 7.5 °C to 12.4 μM after 1 h at 60 °C. Fe was the only metal examined for which the highest (maximum) mobilized concentration was observed at the lowest temperature. When the experiment was terminated after 168 h, the FeDMA concentration had decreased below LOQ in the 60 °C treatment as a result of competitive Fe displacement, whereas in the 7.5 °C treatment mobilized Fe concentrations just began to decrease (to 18.7 μM) and remained in solution by the end of the experiment. Hence, the time span mobilized Fe concentrations were elevated above the LOQ increased with decreasing temperature.

**Fig. 6:**
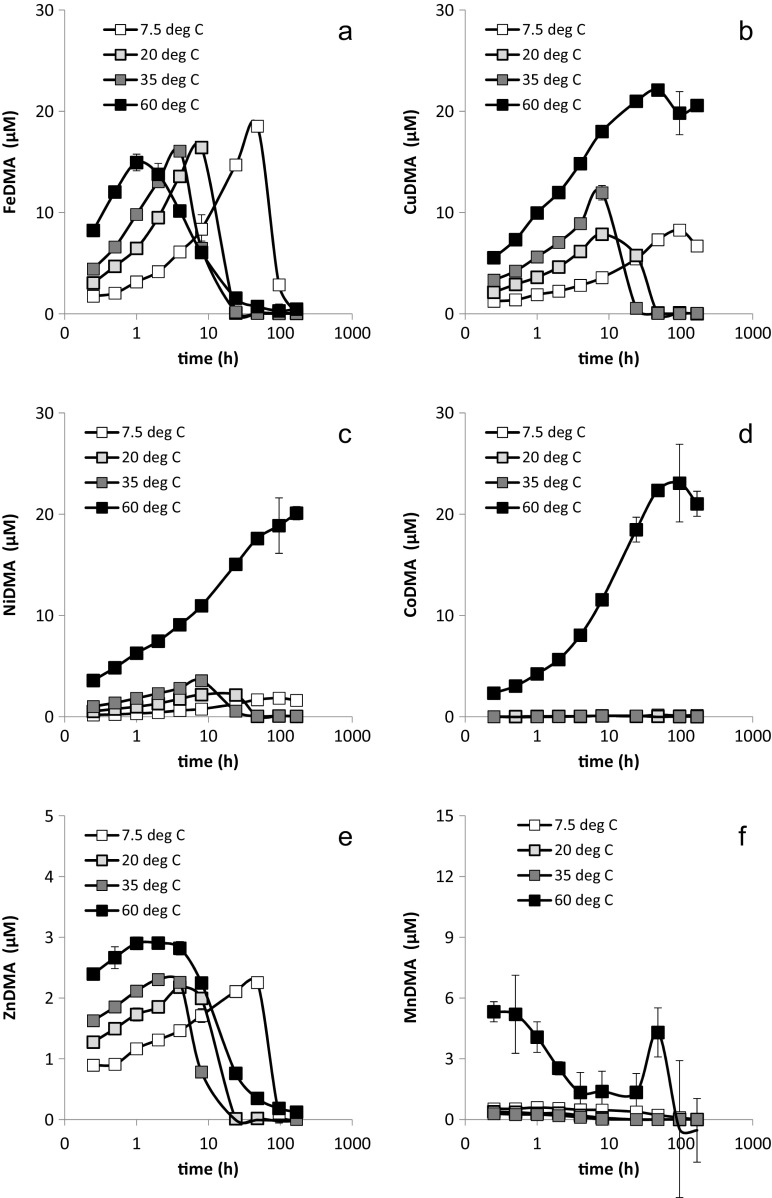
Mobilization of a) Fe, b) Cu, c) Ni, d) Co, e) Zn and f) Mn from by Santomera soil by a 100 μM DMA solution without sterilant (SSR = 1; 10 mM CaCl_2_) at various temperatures. Error bars indicate standard deviations

At 7.5 and 20 °C the solution concentrations of the most important competing metals, Cu, Ni and Co increased throughout the experiment (Fig. 5 b-d); at 35 °C Cu reached a maximum after 96 h and Ni and Co continued to increase, whereas at 60 °C the concentrations of all three elements declined after 96 h. The decline in concentration of all examined metal-DMA complexes after 96 h in the 60 °C treatment is probably not due to biodegradation, because a) sodium azide had been added as sterilant and b) biodegradation appears to be inhibited at this temperature even without addition of sterilant (Fig. 6); thermal degradation of metal-DMA complexes is a more likely explanation. Mobilized Ni and Co concentrations were consistently larger at higher temperature throughout the experiment. After 168 h CuDMA concentrations in the 60 °C treatment dropped below the concentrations in the 35 °C treatment.

For all three metals, the (initial) mobilization rate increased with temperature. Observed maximum mobilized concentrations varied strongly between the temperatures; for Cu it ranged from 11.0 μM at 7.5 °C to 24.3 μM at 35 °C (by a factor 2.2); for Ni from 2.7 μM at 7.5 °C to 18.4 μM at 60 °C (by a factor 6.8); and for Co from 1.7 μM at 7.5 °C to 22.2 μM at 60 °C (by a factor 13.1). Relative differences in mobilized concentrations of Cu, Ni and Co between the temperature treatments were larger for short interaction times than for longer ones.

As ZnDMA is also susceptible to competitive displacement of Zn in Santomera soil, mobilized Zn concentrations evolved similarly as mobilized Fe concentrations: after an initial increase in concentration a maximum was reached, followed by a decrease in concentration (Fig. 5e). With increasing temperature, maximum mobilized Zn concentrations were reached after a shorter interaction time. Particularly at 35 and 60 °C mobilized Zn concentrations reached a maximum after a longer interaction time than mobilized Fe concentrations. Differences in maximum mobilized concentrations were relatively small ranging from 2.5 to 3.2 μM. After 168 h, the ZnDMA concentration in the 60 °C treatment was approaching the LOQ, whereas in the 7.5 °C treatment it had just reached its maximum. Also for Mn a (fast) initial increase in mobilized concentration was observed, followed by a decrease in concentration resulting from competitive displacement, except in the 7.5 °C treatment where the concentration was relatively low and remained approximately constant throughout the experiment (Fig. 5f). The initial increase in MnDMA concentration was larger at higher temperature, and varied by a factor 15.6 (0.7 μM at 7.5 °C and 10.9 μM at 60 °C).

In treatments without azide addition, concentrations of FeDMA, CuDMA, and ZnDMA (Fig. 6) developed similarly over the first 4 h as in the treatment with addition of azide (Fig. 5). For Mn, Co and to a lesser extent Ni, mobilized concentrations differed between treatments with and without azide. Mn and Co mobilization was marginal in treatments at 20 and 35 °C without azide, and the initial mobilized Mn concentration in the 60 °C treatment was halved. Mobilized Ni concentrations were lowered in the 20 and 35 °C treatments without azide.

At 60 °C, no effect of biodegradation on metal mobilization by DMA was observed in the treatments without azide; the decline in the mobilized concentrations of Fe and Zn were exclusively due to competition. Possibly, this temperature was too high for soil microbes to be metabolically active and degrade the DMA ligand. For metals which were mobilized to a substantial degree in all treatments without azide (Fe, Cu, Ni and Zn), the lag time (i.e. the interaction time until microbial activity started to affect metal mobilization by DMA), decreased with increasing temperature from 7.5 to 35 °C. For Fe and Zn it decreased from 48 h (7.5 °C) to 4 h (35 °C), for Cu from 48 h (7.5 °C) to 8 h (35 °C) and Ni from 96 h (7.5 °C) to 8 h (35 °C). The interaction time after which the metal-DMA complexes became depleted from solution also decreased with increasing temperature; for FeDMA after 168 h at 7.5 °C and after 24 h at 35 °C, for ZnDMA after 96 h at 7.5 °C and after 24 h at 35 °C. CuDMA and NiDMA did not become depleted within the time-span of the experiment at 7.5 °C, but were removed from solution after 48 h at 35 °C. Except for the 60 °C treatment, maximum mobilized Fe concentrations were reached one sampling point earlier in the treatments without azide (7.5 °C: 48 h; 20 °C: 8 h and 35 °C: 4 h) than in the corresponding treatment with azide, and differences in maximum concentrations were no larger than 10%. Differences in maximum mobilized Zn, Cu and Ni concentrations were larger, up to 28, 60 and 70%, respectively.

**Fig. 7:**
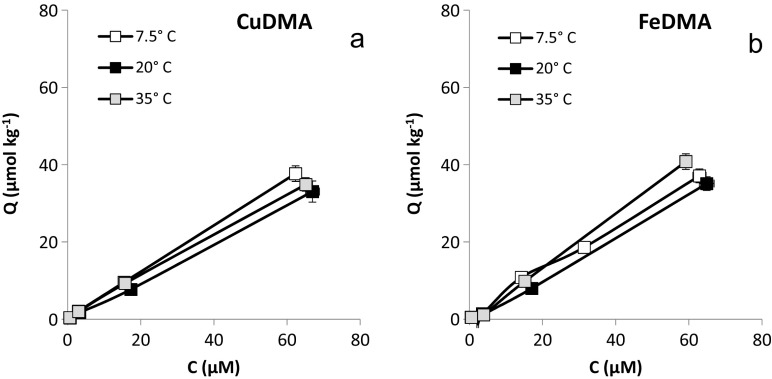
Adsorption isotherms for a) CuDMA and b) FeDMA to Santomera soil at 7.5, 20 and 35 °C. (10 mM CaCl_2_). Error bars indicate standard deviations

The linear adsorption isotherms for FeDMA and CuDMA to Santomera soil (Fig. 7) demonstrate that temperature in the range of 7.5 to 35 °C did not have a pronounced effect on the affinity of metal-DMA complexes for reactive soil surfaces. At all examined temperatures, the slopes of the CuDMA and FeDMA isotherms were similar (Table 3), and no consistent trend of the slope in relation to the temperature was found. The adsorption of the bacterial siderophore ligand DFOB to goethite has been reported to be similarly temperature independent (Cocozza et al. 2002).

**Table 3:** Linear adsorption isotherms for CuDMA and FeDMA to Santomera soil for various temperatures and 10 mM CaCl_2_ (see also Fig. 7)

Species	Temperature (°C)	Linear fit isotherm	R^2^
CuDMA	7.5	0.61*C	1.000
	20	0.49*C	1.000
	35	0.54*C	1.000
FeDMA	7.5	0.60*C	0.993
	20	0.53*C	0.999
	35	0.69*C	1.000

**Fig. 8:**
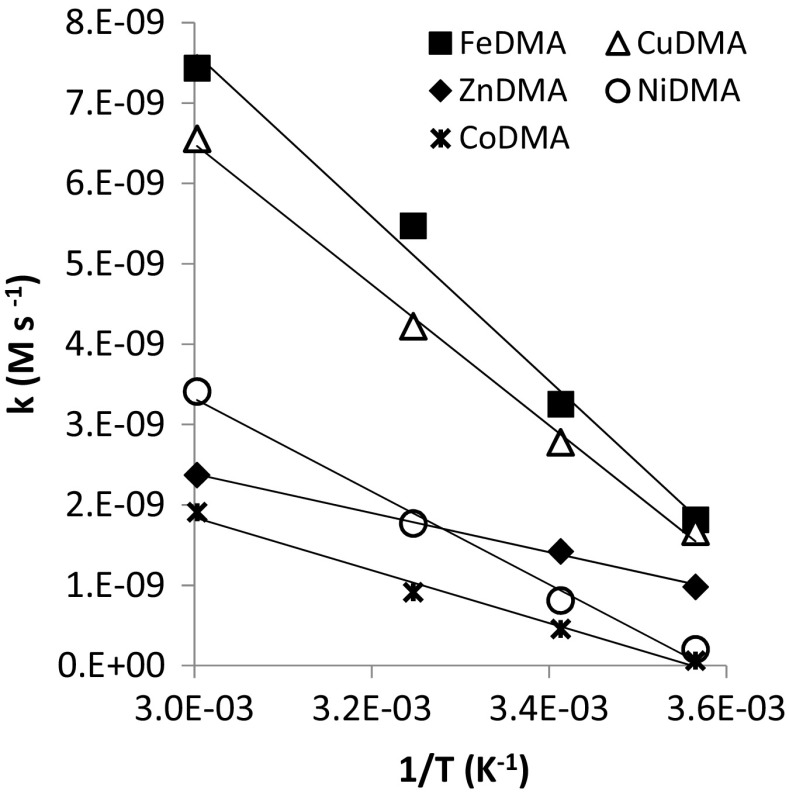
Relation between the initial metal mobilization rate (linearized for the first measured concentration) and the reciprocal of the temperature

Initial metal mobilization rates by DMA from Santomera soil were calculated by dividing the metal-DMA concentration at the first sampling point (0.25 h) by 0.25 h. The calculated rates proved approximately linear with the inverse of the temperature (in degrees Kelvin) (Fig. 8). The slopes of the linear fits provide a measure for the temperature dependence of the mobilization reactions. The rate of Fe mobilization most strongly depended on the temperature, followed by the rates of Cu, Ni, Co and Zn mobilization, respectively. Figure 8 differs from a typical Arrhenius plot, which is used for determining activation energies, with respect to the y-axis where the rate rather than the natural logarithm of the rate is displayed suggesting that the mobilization rates do not follow an Arrhenius type temperature dependence.

## Discussion

The results from the soil interaction experiments provide insights in how metal mobilization by phytosiderophores is affected by rhizosphere pH, electrolytes and temperature. However, from which soil solid phase metals were mobilized remains unclear. In a previous study, equilibrium metal speciation upon interaction of Siebenlinden and Santomera soil with DMA were predicted by multi-surface complexation modeling (Schenkeveld et al. 2014a). This study suggested that in both soils, Cu bound to NOM was the principal Cu pool targeted by DMA, while in Santomera soil, Ni adsorbed to metal(hydr)oxide surfaces was the principal Ni pool. Zn mobilization was predicted to be negligible and for CoDMA no complexation constants were available. For metals like Fe and Mn, of which the activity is governed by mineral solubility, equilibrium modelling predicts metal mobilization from the respective mineral phases. These multi-surface models do not include all reactive compounds present in actual soils and do not account for kinetic considerations. Therefore, spectroscopic investigations are needed to elucidate how phytosiderophores affect metal speciation in soils, and which metal pools are mainly targeted.

### Effect of soil pH

The effect of liming on Fe mobilization by DMA was not straightforward. In the treatments without Cu application, FeDMA strongly dominated the DMA solution speciation; CuDMA accounted for 5% of the DMA, or less (Fig. 1). For most of the experimental duration, mobilized Fe concentrations were considerably higher in the limed treatment. With Cu competition of minor importance and biodegradation inhibited through addition of a sterilant, adsorption is the most obvious process to explain the differences in FeDMA solution concentration.

The change in pH resulting from liming can lead to changes in speciation both in the solid and the solution phase. In the unlimed soil (pH 4.5), the Fe(hydr)oxide surfaces are more positively charged than in the limed soil (pH 6.8), leading to a larger electrostatic attraction between the DMA ligand and the surface. Fe(hydr)oxide minerals are considered an important soil-Fe pool for siderophore-promoted Fe dissolution, and adsorption of the siderophore ligand is a prerequisite step in the ligand-promoted dissolution mechanism (Kraemer 2004). Thus, enhanced adsorption of the DMA ligand to Fe(hydr)oxide surfaces at lower pH may enhance initial Fe mobilization rates and reduce the time required to reach equilibrium. The longer time required for reaching maximum mobilized Fe concentrations in the limed treatment supports that.

The lower maximum mobilized Fe concentration in the non-limed treatment may be explained in several ways. First, in accordance with the complexation constants reported by Murakami et al. (1989), a negatively charged FeOHDMA complex would be predicted as the dominant FeDMA species over the entire soil-relevant pH range. Similarly as the free ligand, this complex would have a higher affinity for Fe(hydr)oxide surfaces which are more positively charged at lower pH, leading to more adsorption to these surfaces. However, Walter et al. (2016) recently reported that negatively charged clay mineral surfaces appear to be the dominant adsorption surface for metal-DMA complexes. The adsorption mechanism is not cleared up yet, but the electrostatic repulsion between a negatively charged metal-DMA complex and the negatively charged clay mineral surfaces is unfavorable for adsorption. von Wiren et al. (2000) demonstrated by high-voltage electrophoresis that the FeDMA complexes undergoes a shift in charge from approximately 0 to −0.6 when the pH was raised from pH 4–5 to pH 7. This suggests that at lower pH, the repulsion between mineral surface and FeDMA complex is smaller, potentially leading to more FeDMA adsorption.

The cause for the gradual decline in FeDMA concentration in the non-limed treatment is unclear. Walter et al. (2016) reported that particularly FeDMA could only partly be desorbed from the soil and that the non-desorbable fraction seemed to increase with time. This suggests that different types of sorption mechanisms may be involved in FeDMA complex adsorption – a reversible and an irreversible one. Possibly, the irreversible sorption process is more gradual, resulting in the slow decline in FeDMA concentration. A similar gradual decline in metal complex concentration related to irreversible sorption has been observed for o,o-CuEDDHA (Schenkeveld et al. 2014c, 2015). The nature of this slow irreversible sorption mechanism, and why this is particularly relevant at low soil pH, needs to be further examined. Other mechanisms that reduce the FeDMA concentration, like thermal degradation, cannot be excluded.

In treatments with Cu addition (Figure 1a2 and b), mobilized Fe concentrations were smaller than in the corresponding treatments without Cu addition as a result of increased Cu availability, which resulted in more competitive Cu complexation and mobilization by DMA. In the non-limed treatment at pH 4.5, the impact from Cu competition remained limited and the CuDMA quickly reached a concentration that remained approximately constant throughout the experiment. In the limed treatment (pH 6.8), the FeDMA concentration first increased, reached a maximum and then decreased. This decrease resulted from competitive Fe displacement by Cu; the CuDMA concentration continued to increase throughout the experiment. Similar observations were done upon DMA application to Santomera soil (Schenkeveld et al. 2014b). This competitive complexation of Cu constrains the size of the window of Fe uptake.

In summary, liming increased the soil-pH and decreased the availability of both Fe and Cu (Table 1), yet the relative extent to which Cu availability decreased was smaller. As a result, competitive complexation and mobilization of Cu increased by liming. In soil batches with low Cu availability (no Cu amendment), the stronger tendency of FeDMA to remain in soil solution at higher soil pH greatly overcompensated the negative effect of enhanced Cu mobilization at higher pH. This resulted in a larger overall Fe mobilization in the lime treated soil batch with low Cu availability, compared to the corresponding non-lime treated batch, falsifying our hypothesis. Cu amendment led to an increase in competitive complexation of Cu by DMA, which had a negative impact on Fe mobilization. The impact was larger at higher pH and decreased the difference in mobilized Fe concentrations between the limed and non-limed soil batch. Extrapolation of this trend suggests that if Cu availability would be further increased, this would eventually lead to less Fe mobilization in the limed treatment compared to the corresponding non-limed treatment. In this scenario our hypothesis of lower Fe mobilization in limed soils would hold. Additionally, in absence of a sterilant, liming appeared to enhance the rate of biodegradation of the DMA ligand, further constraining the window of Fe uptake. So, depending on the potentially available Cu content of the soil, liming can make Fe more or less available to Strategy II plants. Particularly on soils rich in Cu or to which large amounts of Cu are applied as pesticide, liming may have an adverse effect on Fe acquisition by graminaceous crop plants. Inversely, rhizosphere acidification in alkaline and calcareous soils could enhance Fe availability to Strategy II plants, in case the soil has a high potentially available Cu content. If the Cu content is however low, a decrease in rhizosphere pH will have an adverse effect on Fe availability. Fe acquisition and internal re-distribution of iron within strategy II plants may also be influenced by bicarbonate concentrations, which are in turn influenced by soil pH and the presence of solid phase carbonates (Alhendawi et al. 1997). For instance, PS exudation rates of Strategy II plants have been shown to strongly increase with decreasing free Fe^3+^ activity (Fan et al. 1997), which can result from a lime-induced increase in pH. This discussion focusses on rhizosphere chemistry, however, and does not extend to plant physiological processes.

### Effect of type and concentration of the electrolyte

Mobilization of all examined metals by DMA from Santomera soil decreased with increasing electrolyte concentration. For equal ionic strength mobilized concentrations were larger when the electrolyte consisted of the monovalent Na instead of the divalent Ca (Fig. 2). Adsorption of metal-DMA complexes to the soil solid phase increased with increasing electrolyte concentration and was larger for the Ca containing electrolyte (Fig. 3, Table 2).

At the pH of Santomera soil, metal-DMA complexes are negatively charged (von Wiren et al. 2000; Murakami et al. 1989), leading to an electrostatic repulsion between these complexes and negatively charged reactive soil compounds like soil organic matter (SOM) and clay minerals (Walter et al. 2016). With increasing electrolyte concentration, the negative charge of the soil reactive compounds becomes increasingly shielded and the related diffuse double layers shrink in size. As a result, the electrostatic repulsion between metal-DMA complexes and soil surfaces decreases and sorption is enhanced. The decrease in mobilized metal concentrations with increasing electrolyte concentration may hence be explained by a larger fraction of the metal-DMA complexes adsorbing. Furthermore, divalent cations like Ca tend to have a stronger interaction with reactive soil compounds, through specific (chemical) binding than monovalent cations like Na. Through this chemical interaction, positive charge is attributed to the soil reactive compound, increasing its (surface) potential and decreasing the repulsive force towards negatively charged metal-DMA complexes. This decreased repulsion leads to enhanced adsorption compared to treatments with monovalent cations. Ca may also have a bridging role in the formation of ternary complexes between e.g. clay mineral surfaces and negatively charged DMA species (Siebner-Freibach et al. 2006).

The lack of differences in total (solid and solution) CuDMA and initially also FeDMA concentrations between the treatments with different electrolytes (Fig. 4), combined with the differences in solution concentrations (Fig. 2), indicate that the electrolyte did not affect the rate of complex formation, but only the partitioning between the solid and solution phase. This may also hold for other metal-DMA complexes like NiDMA, CoDMA and ZnDMA, but needs to be further investigated by means of adsorption isotherms.

At Santomera soil pH also the free DMA ligand has a negative charge, and therefore electrolyte type and concentration would be expected to affect adsorption of the free ligand in a similar way as adsorption of metal-DMA complexes. Free ligand concentrations have not been determined to establish this. Assuming that adsorption of the free ligand is indeed enhanced at higher electrolyte concentration, the enhanced adsorption does not result in enhanced Cu or Fe complexation rates (Fig. 4). This seems to imply that either complexation of these metals is not surface controlled or that adsorption predominantly takes place at surfaces other than those where these metals are complexed, and that adsorption of the free DMA ligand to these latter surfaces is not affected by the electrolyte.

Our observations suggest that electrolyte type and concentration mainly affect the window of Fe uptake through adsorption of FeDMA complexes, confirming our hypothesis. At higher electrolyte concentrations and in the presence of divalent instead of monovalent cations, adsorption of the FeDMA complex is enhanced, resulting in a lower FeDMA solution concentration and hence a smaller Fe uptake window. The smaller mobilized concentrations of labeled Fe by PS from a calcareous soil at higher NaCl background concentrations observed by Awad et al. (1988) are consistent with this. Therefore, plants grown on saline soils or on soils receiving a high anthropogenic salt input, e.g. through irrigation (Pitman and Lauchli 2002) may need to exude more PS than plants grown on comparable non-alkaline soils for meeting their Fe requirements. As a consequence, high salt concentrations may induce micronutrient (e.g. Fe) deficiency stress in plants in addition to salinity stress. Grasses may have partly adapted to the effect salinity has on the efficiency of Strategy II Fe acquisition by increased PS exudation, as observed in hydroponic systems (Daneshbakhsh et al. 2013) and in soils (Oburger et al. 2014).

### The effect of temperature

Upon interaction of DMA with soil, the initial mobilization rates of both Fe and competing metals increased with temperature. Once the free ligand started to become depleted, the decline in FeDMA concentration was enhanced at higher temperature as a result of displacement of Fe by competing metals. The overall reaction rates of the mobilization and displacement reactions are determined by the rate-limiting steps in the respective reaction mechanism. Hence, the influence of temperature on these rate-limiting steps are decisive for the overall rates. In model systems it was observed that for ligand-promoted dissolution of minerals, the detachment of the metal ion forming a surface complex with the ligand from the crystal lattice is generally considered the rate-limiting step (Stumm 1993). Complexation of SOM-bound metals involves a ligand exchange mechanism. However, the current understanding and quantitation of the reaction mechanisms involved with metal complexation and mobilization by PS from soil is limited. Further detailed investigations of these mechanisms are required for a mechanistic interpretation of the results presented here.

The biodegradation rate of the PS ligand was also affected by temperature. Among the temperatures examined the rate had a maximum at 35 °C; at 60 °C no biodegradation was observed, possibly due to metabolical inactivation of soil microorganisms at exceptionally high soil temperatures. Additionally, the lag phase until PS speciation became affected by degradation decreased with increasing temperature. Inhibiting microbial activity also affects microbial Mn-redox cycling and concentrations of Mn and other metals that are associated with Mn-oxide minerals (e.g. Ni and Co) (Schenkeveld et al. 2017; Sparrow and Uren 2014). These effects on the window of Fe uptake are however small in comparison to microbial degradation of the PS ligand.

As hypothesized in the introduction, temperature affects the window of Fe uptake mainly through the rates of complexation of Fe and competing metals and Fe displacement from FeDMA, as well as the rate of biodegradation. The effect of temperature on adsorption of DMA species appears to be small within the range of environmentally relevant temperatures. Agronomically important graminacious crops are grown in different climatic settings and seasons around the globe. Plants grown at higher temperatures (in warm climatic regions or in warm seasons) may increase Fe availability faster upon exudation of PS. At higher temperature also evapotranspiration is higher (assuming a similar soil water potential), facilitating advective transport of the FeDMA complexes to the roots surface and allowing the plant to more readily take up the mobilized Fe. Possibly, the faster increase in Fe mobilization and the faster transport enable plants to cope with the faster competitive displacement of Fe from FeDMA complexes and the faster biodegradation of the DMA ligands at higher temperature. Despite the slower Fe mobilization at lower temperatures (in cold climatic regions or cold seasons), the later onset of Fe displacement from FeDMA, the lower Fe displacement rate and the lower biodegradation make that the window of Fe uptake is considerably larger at lower temperatures. Possibly this larger window enables plants to deal with a smaller advective transport-mass flux of Fe towards to root surface.

## Electronic supplementary material

ESM 1 (DOCX 146 kb)
