# Supplementary material for: The effect of pH, electrolytes and temperature on the rhizosphere geochemistry of phytosiderophores
Source: Plant Soil. 2017 Apr 13;418(1):5–23. doi: 10.1007/s11104-017-3226-9 (PMC5605604; doi:10.1007/s11104-017-3226-9)
Supplement: Supplementary file 1 — (DOCX 146 kb) [file 11104_2017_3226_MOESM1_ESM.docx]

*Supporting Information for the publication: The effect of pH, electrolytes and temperature on the rhizosphere geochemistry of phytosiderophores*

M. Walter ^a^, S.M. Kraemer^a*^, W.D.C. Schenkeveld^a*^

^a^ University of Vienna

Dept. of Environmental Geosciences Center for Earth Sciences

Althanstraße 14 (UZA II)

1090 Vienna, Austria

T: +43-1-4277-531 42

*Email: [walter.schenkeveld@univie.ac.at](mailto:walter.schenkeveld@univie.ac.at); [stephan.kraemer@univie.ac.at](mailto:stephan.kraemer@univie.ac.at)

**SI-Figure 1:** Sub-micromolar Zn concentrations mobilized by a 30 µM DMA at 0 and 5 mg kg^-1^ Cu and 0% (pH=4.5) and 0.5% (pH=6.8) CaCO₃ added to Siebenlinden soil. Zn mobilization was not influenced by addition of 5 mg kg^-1^ Cu, but rather by soil pH.

**b**

a

**SI-Figure 2:** Modelled DMA speciation as a function of pH a) in presence of 110 µM Mn^2+^(aq) and 30 µM DMA (10 mM CaCl_2_ as background electrolyte) and b) in presence of 0.1 M Ca^2+^ and 30 µM DMA under atmospheric CO_2_ partial pressure (400 ppm) allowing aragonite (CaCO_3_) to precipitate. Modelling was done in ECOSAT.

**SI-Figure 3:** a) Fe and b) Cu concentrations mobilized by 30 µM DMA from Siebenlinden soil amended with 0, 0.5 and 5 wt% CaCO₃; 10 mM CaCl_2_ and 0.2 g l^-1^ bronopol Apart from a small difference in pH between the 0.5 and 5 wt% CaCO₃ treatment (pH=6.8 and pH=7.2), no relevant difference in Fe and Cu mobilization by DMA was observed.

**SI-Figure 4:** a) Fe and b) Cu concentrations mobilized by 30 µM DMA from 0 and 0.5 CaCO₃ wt% amended Siebenlinden soil with and without addition of sterilant (0.2 g l^-1^ Bronopol). Biodegradation of FeDMA and CuDMA was slightly influenced by addition of 0.5 wt% lime: in the non-limed treatments FeDMA and CuDMA concentrations decreased below the LOQ after 48h, in the limed treatments after 96h of interaction.

**SI-Table 1:** Mean Ca and Na concentrations over all interaction times for the treatments in the “electrolyte experiment”. Additionally, the pH range and the minimum and maximum difference in pH value between the UPW and the other electrolyte treatments are listed.

| Treatment | Ca concentration [mM] | Na concentration [mM] |  | pH range | Minimum and maximum pH difference relative to UPW treatment |
| --- | --- | --- | --- | --- | --- |
| UPW | 1.5 | 0.32 |  | 7.8-8.1 |  |
| 2 mM CaCl₂ | 2.9 | 0.44 |  | 7.4-8.0 | 0.0-0.4 |
| 10 mM CaCl₂ | 9.1 | 0.47 |  | 7.4-7.8 | 0.2-0.6 |
| 100 mM CaCl₂ | 91.6 | 0.56 |  | 7.1-7.4 | 0.6-0.9 |
| 300 mM NaCl | 19.7 | 234 |  | 7.5-7.7 | 0.2-0.5 |

| Time[h] | CuDMA | | | | NiDMA | | | | CoDMA | | | |
| --- | --- | --- | --- | --- | --- | --- | --- | --- | --- | --- | --- | --- |
|  | UPW/2mM CaCl₂ | UPW/10mM CaCl₂ | UPW/100mM CaCl₂ | UPW/300mM NaCl | UPW/2mM CaCl₂ | UPW/10mM CaCl₂ | UPW/100mM CaCl₂ | UPW/300mM NaCl | UPW/2mM CaCl₂ | UPW/10mM CaCl₂ | UPW/100mM CaCl₂ | UPW/300mM NaCl |
| 0.25 | 1.1 | 1.2 | 1.5 | 1.1 | 1.2 | 1.3 | 1.9 | 1.3 | 0.8 | 1.5 | 1.7 | 1.6 |
| 0.50 | 1.1 | 1.2 | 1.4 | 1.1 | 1.0 | 1.2 | 1.8 | 1.4 | 1.1 | 2.4 | 2.9 | 2.0 |
| 1 | 1.1 | 1.3 | 1.4 | 1.1 | 1.1 | 1.4 | 1.8 | 1.5 | 1.0 | 1.5 | 2.2 | 1.7 |
| 2 | 1.0 | 1.2 | 1.4 | 1.1 | 1.1 | 1.4 | 1.9 | 1.5 | 1.1 | 1.7 | 2.7 | 1.9 |
| 4 | 1.1 | 1.3 | 1.5 | 1.1 | 1.1 | 1.4 | 1.9 | 1.5 | 1.0 | 1.5 | 2.1 | 1.5 |
| 8 | 1.0 | 1.2 | 1.4 | 1.1 | 1.1 | 1.4 | 1.8 | 1.5 | 1.1 | 1.5 | 2.3 | 1.8 |
| 24 | 1.0 | 1.2 | 1.4 | 1.1 | 1.1 | 1.3 | 1.8 | 1.5 | 1.2 | 1.5 | 2.2 | 1.8 |
| 48 | 1.1 | 1.2 | 1.4 | 1.2 | 1.1 | 1.4 | 1.9 | 1.5 | 1.1 | 1.4 | 2.0 | 1.7 |
| 96 | 1.0 | 1.2 | 1.4 | 1.1 | 1.1 | 1.4 | 1.8 | 1.5 | 1.1 | 1.4 | 2.0 | 1.7 |
| 168 | 1.1 | 1.2 | 1.3 | 1.1 | 1.1 | 1.3 | 1.8 | 1.4 | 1.0 | 1.2 | 1.8 | 1.6 |

**SI-Table 2:** Concentration ratios of mobilized Cu, Ni and Co by a 100 mM DMA as a function of interaction time. The ratios were calculated by dividing the concentration of the ultra-pure water (UPW) treatment by the concentrations of the other electrolyte treatments. For Cu and Ni the ratio remained approximately constant over all time points examined. For Co the ratios became constant only after approximately 4 h; this is related to the relatively small Co mobilization in the first few hours, and the large relative error in the measured concentrations.
